# Simulated natural daylight and twilight modulate activity and light sampling behaviour in mice

**DOI:** 10.1186/s12915-026-02517-7

**Published:** 2026-01-22

**Authors:** Laura C. E. Steel, Mark W. Hankins, Russell G. Foster, Stuart N. Peirson

**Affiliations:** Sir Jules Thorn Sleep and Circadian Neuroscience Institute (SCNi), Kavli Institute for Nanoscience Discovery, Nuffield Department of Clinical Neurosciences, University of Oxford, Dorothy Crowfoot Hodgkin Building, Oxford, United Kingdom

**Keywords:** Circadian rhythms, Photoentrainment, Intensity, Spectrum, Twilight, Daylight, Light sampling behaviour, Photoreceptors, Circadian ecology

## Abstract

**Background:**

In the wild, mice are subject to changes in light intensity and spectrum (colour) across the solar day. In addition, mice are able to self-modulate their light exposure – a concept termed light sampling behaviour, which results in intermittent patterns of light exposure. These complexities are poorly considered in most laboratory animal housing. As such, our understanding of the role of intermittent exposure to naturally-occurring changes in intensity and spectrum in circadian behaviour are limited. To address these issues we simulated both daylight and twilight in the laboratory, and provided a dark nestbox to enable behavioural regulation of light exposure.

**Results:**

The results show that gradual changes in light intensity are a key driver of crepuscular light sampling in mice, whilst demonstrating for the first time that spectral cues at twilight modulate the timing of behaviour – advancing locomotor activity by 0.5h and light sampling behaviour by 1.1h.

**Conclusions:**

Collectively, our results demonstrate the significance of changes in intensity and spectrum across twilight for regulating mouse behaviour. These findings highlight important differences in mouse behaviour under naturalistic environments compared to normal laboratory conditions.

**Supplementary Information:**

The online version contains supplementary material available at 10.1186/s12915-026-02517-7.

## Background

To anticipate predictable changes in the environment and increase fitness, organisms must align their internal circadian clock to the external environment [1–3]. This is achieved using external time-cues (zeitgebers). In mammals the primary zeitgeber is light, via a process known as photoentrainment [4]. The natural light environment is highly complex and dynamic compared to that of the laboratory. In particular, predictable changes in intensity and spectrum occur across the 24 h day [5–7], and animals are also able to self-regulate their light exposure via light sampling behaviour [8–13]. It is well established that changes in the intensity and timing of light exposure play a key role in photoentrainment [4]. In addition, spectral changes [14–19] and intermittent patterns of light exposure resulting from light sampling [9, 12, 20] have also been shown to contribute to photoentrainment.

The greatest changes in intensity and spectrum occur at twilight (dawn and dusk). Perhaps unsurprisingly, the circadian clock is most sensitive to light at this time [21–24]. Our recent data demonstrates that twilight is also when mice show highest levels of light sampling behaviour in the presence of a nestbox [12]. Twilight is separated into three phases as defined by solar elevation (civil twilight, 0 to −6 degrees; nautical twilight, −6 to −12 degrees; astronomical twilight, −12 to −18 degrees) and varies in length with time of year and latitude. Light intensity can increase from ~ 0.001 photopic lux at astronomical twilight to 1000 photopic lux at civil twilight, reaching > 100,000 photopic lux on a sunny day [5, 25]. Cloud cover and moonlight provide further variation in irradiance levels [6, 7, 15, 26, 27]. With regard to spectrum, the spectral power distribution (SPD) of daylight has a peak power of ~ 460nm [28, 29], which becomes progressively short-wavelength enriched (< 500nm) across twilight [5, 7, 19]. This is due to an increase in atmospheric absorption and scatter—primarily of the Chappius effect [30].

In contrast to the complexities of the natural light environment, laboratory studies are generally performed under 12:12h light/dark (LD) cycles of broad spectrum white light sources, which are typically short-wavelength depleted compared to daylight and twilight [31]. Whilst the use of short-duration (1-2h) ramped intensity LD cycles is common in animal facility holding rooms, spectral changes are still lacking. Furthermore, rodent species rarely have the opportunity to self-select their light exposure [8, 9, 11, 12]. Our recent research showed that when given the choice, mice only expose themselves to 0.8h of light across a 12 h day (12:12h LD cycle), primarily at twilight [12]. It has been suggested that simplified laboratory conditions, whilst extremely informative, may be responsible for the differences in circadian behaviour frequently observed between laboratory and natural environments in rodents [13, 32–37]. Over-simplification of environmental conditions in the laboratory may limit our understanding of circadian entrainment to a set of specific, artificial conditions [38–42]. Whilst differences in the timing of behaviour under natural conditions could be driven by multiple factors including food availability, temperature, predation and social structure, the importance of light in mammalian photoentrainment suggest it is likely to be a significant factor.

The depleted short-wavelength (< 440nm) components of laboratory white light sources—such as white LEDs—may be particularly relevant for non-image forming (NIF) behaviours in animals with ultraviolet sensitive cones, such as mice [43–47]. The mouse retina contains four classes of photoreceptor with differing spectral sensitivities – rods (λ_max_ = 498 nm), short-wavelength sensitive cones (S-cones, λ_max_ = 360 nm), medium wavelength sensitive cones (M-cones, λ_max_ = 508 nm), as well as melanopsin-expressing photosensitive retinal ganglion cells (pRGCs) (λ_max_ = 480 nm). Indeed, differences in the shape of the phase response curve (PRC) under artificial white lights with varying levels of UV light, and daylight, have been shown in rodents [48] and bats [49, 50]. Recent guidance has therefore recommended that laboratory animals should be housed under light environments more closely replicating daylight – the natural conditions under which they evolved. However, more research on the effects of natural light environments on behaviour is needed [31].

**Fig. 1:**
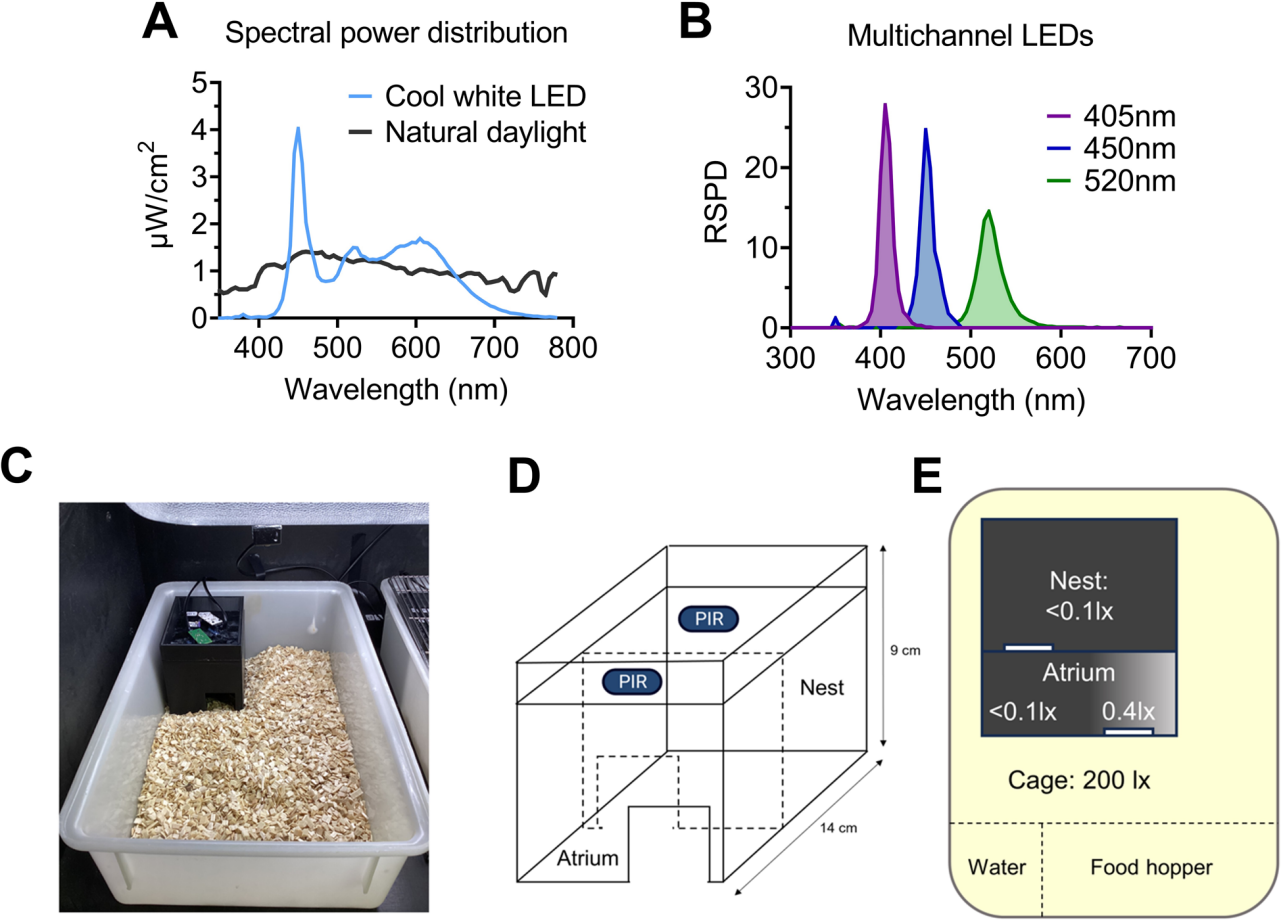
Experimental setup. **A** Spectral power distribution (SPD) of cool white LED (blue) and natural daylight (grey) at 200 photopic lux (170 melanopic lux). Natural daylight measurement taken from Spitschan et al., 2016. **B** Relative SPD (RSPD) of multichannel LEDs (λmax = 405nm (violet); λmax = 450nm (blue); λmax = 520nm (green)). **C** Photo of nestbox in-situ with passive infrared sensor (PIR) above cage. **D** Schematic of nestbox design (not to scale), with PIRs. (E) Light levels across nestbox and cage, measured using an XL-500 BLE Spectroradiometer (NanoLamda, Korea), with location of water bottle and food hopper marked. Panels C-E reproduced from [12]

Twilight is a key signal for photoentrainment. This is evidenced by skeleton photoperiods (which deliver brief light exposure at dawn and/or dusk only) being sufficient for entrainment in rodents [51, 52] and successfully encoding daylength in the master circadian pacemaker located in the hypothalamic suprachiasmatic nuclei (SCN) [53]. Differences at twilight in natural and laboratory light environments may therefore be particularly relevant for explaining behavioural differences between these conditions. There are several cues associated with natural twilights which could be used to tell the time of day—changes in intensity, changes in spectrum, and the position of the sun relative to the horizon [6, 14, 23, 54]. Changes in intensity are known to regulate circadian entrainment [55], whilst changes in spectrum have only recently been explored in mammals [15, 19]. Tracking twilight using spectral cues would require colour detection, which involves the comparison of signals from two or more photoreceptors with different spectral sensitivities (known as spectral opponency; [56, 57]. Recent theoretical studies have demonstrated that spectral changes across twilight would provide a reliable indicator of solar time, and could be tracked using spectral opponency [18, 27]. In parallel, spectral opponency in mouse SCN neurons has been identified, and differences in circadian entrainment under natural twilights have been observed, characterised by a delay in body temperature rhythms [19]. Here we study how simulated natural daylight and twilight changes in intensity and spectrum influence patterns of locomotor activity and light sampling behaviour in mice [12] compared to standard laboratory lighting (Fig. 1A, blue line). By using multiple different wavelength LEDs (Fig. 1B) we simulate the experience of daylight and twilight to the mouse visual system, in the presence of a dark nestbox (Fig. 1C-E) to enable animals to self-select their light exposure [12].

## Results

### Nestbox use under a white LED is consistent with previous studies

**Fig. 2:**
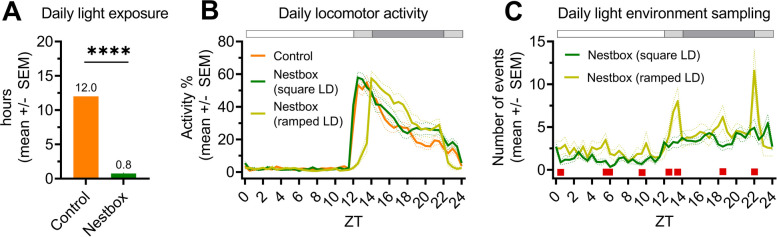
Nestbox use is consistent with previous studies. **A** Daily light exposure (hrs) under a square-wave LD cycle without a nestbox (control; orange) and with a nestbox (green). **** *p* < 0.001 between condition comparisons. **B** Main cage daily locomotor activity profile under a square-wave LD cycle without a nestbox (control; orange) and with a nestbox (green), and under a ramped LD cycle with a nestbox (yellow). **C** Daily light environment sampling profile under a square-wave LD cycle (green) and a ramped LD cycle (yellow). Red squares indicate significant post hoc differences between groups. (A,B,C) LD cycle of a cool white LED used in all conditions. Square-wave LD cycle refers to 12:12h LD. Ramped LD cycle refers to 12:2:8:2h LD cycle. ZT refers to “zeitgeber time” – with ZT0 defined as lights fully on. White, grey and black bar shows timing of light, light ramp and dark, respectively. All results reported as mean across mice and days, ± SEM

Passive infrared sensors (PIRs) were used to monitor locomotor activity every 1 s across the main cage and both sections (atrium and nest) of the nestbox. From this, light sampling behaviour was quantified – defined as a movement from the nest to atrium section of the nestbox (details outlined in the ‘ Locomotor activity monitoring’ Methods section). As described in [12] the majority of mice (11/12) used the nestbox (Additional file 1: Fig.S1A), reducing their daily light exposure to an average of 0.8h across the 24 h period (Fig. 2A). This is significantly lower than the control condition [t(10) = 86.3, *p* < 0.0001; one-sample t-test against a control mean of 12.0h]. Daily light exposure is significantly lower in males compared to females (Additional file 1: Fig.S1B) [t(12) = 10.2, *p* < 0.0001, unpaired t-test]. This matches the higher levels of main cage locomotor activity in females than males during the light phase in both the control and nestbox conditions under a square-wave LD cycle [main effect of sex, F(1,10) = 6.3, *p* = 0.0307; but no significant condition x sex interaction; three-way repeated measures ANOVA; data not shown]. This could result from higher oestrogen levels in females, which promotes CNS arousal [58]. Daily locomotor activity (Fig. 2B) and light environment sampling profiles (Fig. 2C) show similar patterns to those in [12] with a 12:2:8:2h ramped LD cycle generating crepuscular peaks of light environment sampling that are not observed under a 12:12h square-wave LD cycle (Fig. 2C) [main effect of time, F(6.2,67.9) = 12.7, *p* < 0.0001; no main effect of condition, F(1,11) = 1.4, p = 0.2576; time x condition, F(6.0,65.5) = 3.6, *p* = 0.0038; two-way repeated measures ANOVA]. This latter interaction demonstrates the difference in pattern of light environment sampling behaviour across time between conditions, including at dawn and dusk as expected [post-hoc differences were observed at dawn (ZT13; *p* = 0.0337) and dusk (ZT22; *p* = 0.0328); Fisher’s LSD]. ZT refers to “zeitgeber time” – with ZT0 defined as lights fully on. Overall, nestbox use under a square-wave and ramped LD cycle of white LED in this study is comparable to that of [12].

### Replacing a square-wave LD cycle of white LED with simulated daylight has subtle effects on behaviour

**Fig. 3:**
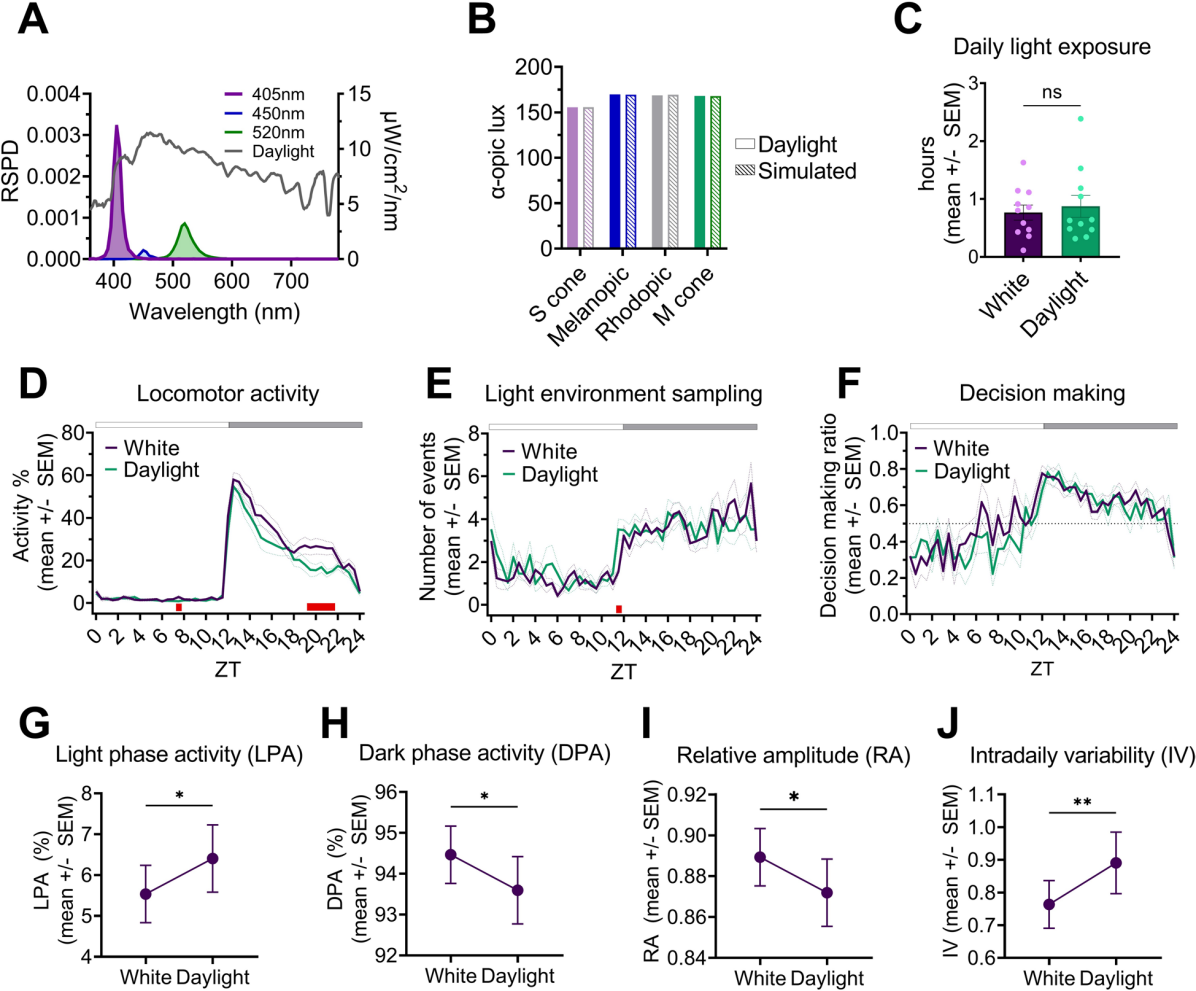
Simulation of, and behaviour under, a 12:12h square-wave LD cycle of cool white LED (‘White’) and a 12:12h square-wave LD cycle of simulated daylight (‘Daylight’). **A** RSPD of daylight (grey, data from Spitschan et al., 2016) on left y-axis. The power (μW/cm^2^/nm) of each multichannel LED (λmax = 405nm (violet); λmax = 450nm (blue); λmax = 520nm (green)) required to simulate daylight (grey) on right y-axis. **B** α-opic lux produced by 200 photopic (170 melanopic lux) of daylight (data from Spitschan et al., 2016) and simulated daylight (‘simulated’) (produced by the LEDs in panel A). **C** Daily light exposure (hrs) with nestbox present, under white and simulated daylight conditions. **D** Main cage daily locomotor activity profile with nestbox present, under white and simulated daylight conditions. **E** Daily light environment sampling profile with nestbox present, under white and simulated daylight conditions. **F** Daily decision making profile with nestbox present, under white and simulated daylight conditions. Values > 0.5 indicate more ‘go’ decisions than ‘no-go’. (D,E,F) White and black bar shows timing of light and dark, respectively. Red squares indicate significant post hoc differences between groups. **G-J** Key circadian parameters under white and simulated daylight conditions. ** *p* < 0.01, * *p* < 0.05 between condition comparisons. **(G)** Light phase activity (%). **H** Dark phase activity (%). **I** Relative amplitude. **J** Intradaily variability. ZT refers to “zeitgeber time” – with ZT0 defined as lights fully on. All results reported as mean across mice and days, ± SEM

Changing the square-wave LD cycle from a white LED to the equivalent melanopic lux of simulated daylight (Fig. 3A,B) had modest effects on behaviour. It had no significant effect on the extent of nestbox use and subsequent light exposure (Fig. 3C) [*p* = 0.7646, Wilcoxon signed rank test]. However, main cage locomotor activity over 24 h was significantly lower under simulated daylight than the white LED (Fig. 3D) [main effect of time, F(3.0,32.7) = 65.6, *p* < 0.0001; main effect of condition, F(1,11) = 26.8, *p* = 0.0003; time x condition, F(4.8,52.8) = 4.8, *p* = 0.0013; two-way repeated measures ANOVA with post-hoc Bonferroni test]. This was only true for the dark phase [main effect of condition, F(1,11) = 28.2, *p* = 0.0002], not the light phase [main effect of condition, F(1,11) = 1.2, *p* = 0.3039]; likely due to the low overall levels of activity during the light phase.

Regarding light environment sampling behaviour, no significant difference across 24 h between conditions was observed (Fig. 3E) [main effect of time, F(4.9,53.5) = 12.7, *p* < 0.0001; main effect of condition, F(1,11) = 0.1634, *p* = 0.6938; time x condition interaction, F(7.0,77.4) = 1.6, *p* = 0.1344; two-way repeated measures ANOVA]. Furthermore, there was no significant difference in the onset of light environment sampling behaviour between light conditions (paired t-test, data not presented). Similarly, there was no significant effect of condition, or condition x time interaction, on the ratio of decision making (go: no-go decisions) between light conditions (Fig. 3F). However, as expected from the lower dark phase locomotor activity under simulated daylight than the white LED (Fig. 3D), light phase activity (%) (Fig. 3G) was significantly higher under daylight [t(11) = 2.3, *p* = 0.0431], whilst dark phase activity (%) (Fig. 3H) [t(11) = 2.3, *p* = 0.0431] and relative amplitude (Fig. 3I) were both significantly lower [t(11) = 2.3, *p* = 0.0431] (paired t-tests). In addition, intra-daily variability – a measure of activity fragmentation, was significantly higher under simulated daylight than the white LED (Fig. 3J) [t(11) = 3.1, *p* = 0.0094; paired t-test]. These metrics indicate less robust rhythms under simulated daylight compared to the white LED conditions [59]. Other metrics of circadian disruption were calculated (inter-daily stability, period and periodogram power), but no significant differences were observed (data not presented). In summary, replacing a square-wave LD cycle of white LED light with simulated daylight significantly lowered dark phase activity levels (Fig. 3D) and reduced related measures of daily rhythm robustness (Fig. 3G-J), but beyond this had little effect on the behavioural parameters measured.

### Mice show crepuscular peaks in light environment sampling behaviour under a ramped LD cycle of both a white LED and simulated daylight

**Fig. 4:**
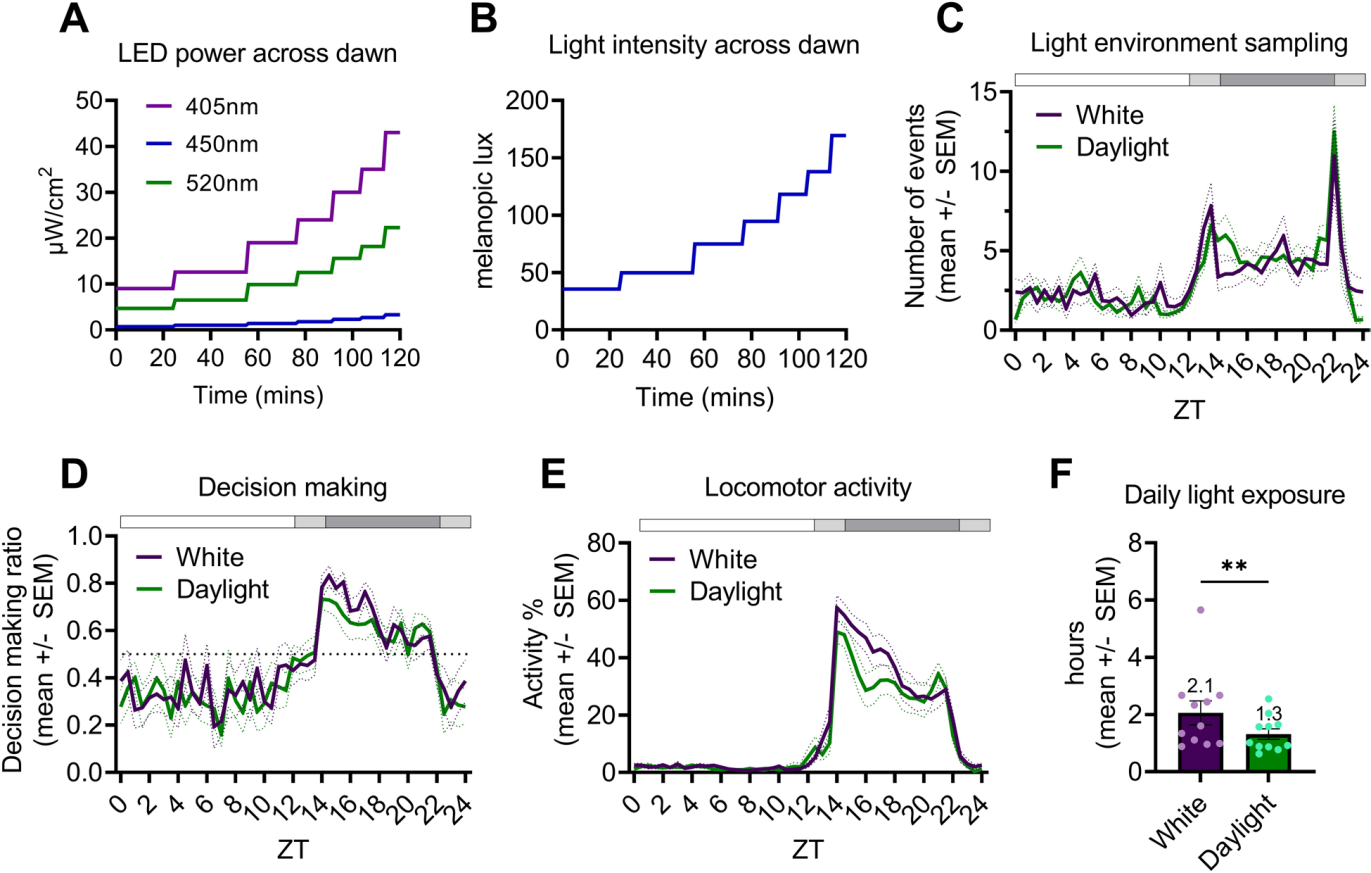
Simulation of, and behaviour under, a 12:2:8:2h LD cycle of cool white LED (‘white’) and a 12:2:8:2h LD cycle of simulated daylight (‘daylight’) (intensity-only ramp).** A** Power (μW/cm^2^/nm) of each multichannel LED (λmax = 405nm (violet); λmax = 450nm (blue); λmax = 520nm (green)) required to maintain a constant α-opic lux ratio, whilst changing intensity, across the 2 h dawn ramp of the simulated ‘daylight’ condition. **B** Changes in light intensity (melanopic lux) across the 2 h dawn ramp. **C** Daily light environment sampling profile with nestbox present, under white and daylight ramped conditions. **D** Daily decision-making profile with nestbox present, under white and daylight ramped conditions. Values > 0.5 indicate more ‘go’ decisions. **E** Main cage daily locomotor activity profile with nestbox present, under white and simulated daylight ramped conditions. **F** Daily light exposure (hrs) under the white and daylight ramped conditions. Light phase defined as start of ramp on to lights fully off. **(C,D,E)** White and black bar shows timing of light and dark, respectively. ZT refers to “zeitgeber time” – with ZT0 defined as lights fully on. All results reported as mean across mice and days, ± SEM. ** *p* < 0.01 between condition comparisons. Red squares indicate significant post hoc differences between groups

Multichannel LEDs were used to generate a 12:2:8:2h ramped LD cycle of simulated daylight, which changed only in intensity across dawn and dusk (simulated “daylight ramp” condition) (Fig. 4A,B). Mice show similar changes in behaviour (light environment sampling behaviour (Fig. 4C), decision-making (Fig. 4D) and locomotor activity (Fig. 4E)) when changing from a 12:12h square-wave to a 12:2:8:2h ramped LD cycle of both white LED (Fig. 2B,C) and simulated daylight (Fig.S3). Under a ramped LD cycle of both white LED (Fig. 2C) and simulated daylight (Fig.S3), mice show clear crepuscular peaks in light environment sampling (Fig. 4C) and there is no significant effect of condition on light environment sampling behaviour (Fig. 4C), or subsequent decision-making behaviour (Fig. 4D). This indicates that it is the ramping of an LD cycle (artificial or natural) that is most important for driving crepuscular light environment sampling behaviour [12].

However, consistent with the lower levels of dark phase activity observed under a square-wave LD cycle of simulated daylight compared to a white LED (Fig. 3D), there is a significant effect of light condition on locomotor activity under a ramped LD cycle of simulated daylight and white LED (Fig. 4E) [main effect of time, F(1.7,18.4) = 54.5, *p* < 0.0001; main effect of condition, F(1,11) = 6.4, *p* = 0.0284; time x condition, F(3.8,41.4) = 4.6, *p* = 0.0044; two-way repeated measures ANOVA with post-hoc Bonferroni test]. As under the square-wave LD cycle conditions (Fig. 3D), this is only significant during the dark phase (ZT14-22) [main effect of condition, F(1,11) = 5.0, *p* = 0.0478] and not the light phase (ZT22-14) [no main effect of condition, F(1,11) = 0.9, *p* = 0.3655]; emphasising it is exposure to simulated daylight during the light phase that is influencing dark phase activity. Despite no significant differences in light phase (ZT22-14) activity levels between conditions, main cage locomotor activity is lower at dusk under the simulated daylight ramp (Fig. 4E). This could explain the significantly lower levels of light exposure by mice under the ramped LD cycle of simulated daylight than white LED—1.3h versus 2.1h, respectively (Fig. 4F) [*p* = 0.0020; Wilcoxon signed rank test].

Overall, changing the light environment from a ramped LD cycle of a white LED to simulated daylight significantly reduces dark phase activity and daily light exposure, but otherwise has modest effects on behaviour. It is the ramping of either LD cycle condition which generates crepuscular peaks in light environment sampling behaviour [12], with spectrum playing a modulating role.

### Introducing spectral changes associated with twilight changes the timing of behaviour at dusk but not dawn

**Fig. 5:**
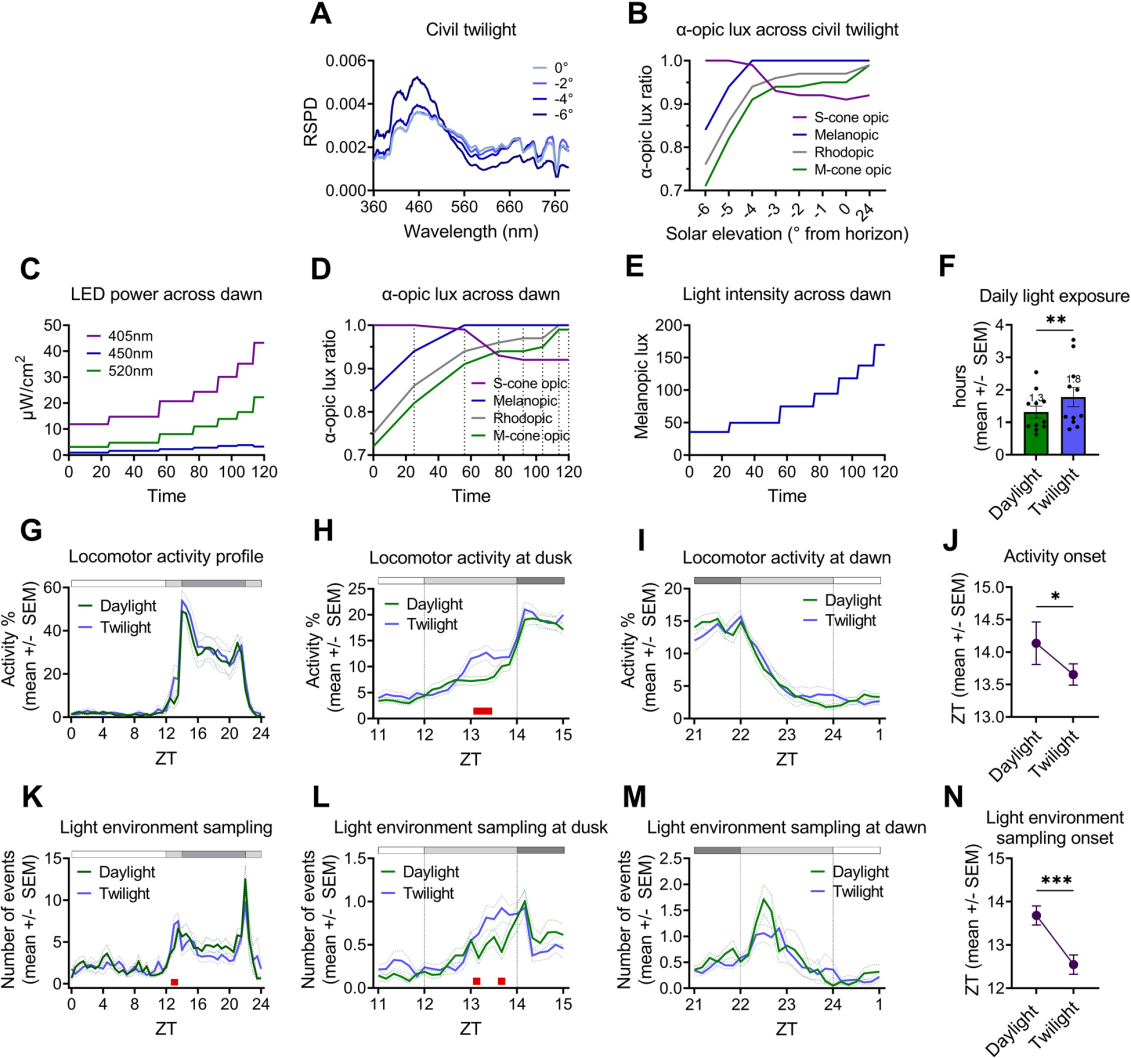
Simulation of, and behaviour under, a 12:2:8:2h LD cycle of simulated daylight (‘daylight’) (intensity-only ramp) and a 12:2:8:2h LD cycle of simulated twilight (‘twilight’) (intensity and spectral ramp).** A** Spectral changes (RSPDs) across civil twilight in increments of 2 degrees of solar elevation, relative to the horizon (data from Spitschan et al., 2016). **B** α-opic lux ratio across civil twilight (relative to the maximum value). **C** Power (μW/cm.^2^/nm) of each multichannel LED (λmax = 405nm (violet); λmax = 450nm (blue); λmax = 520nm (green)) required to produce both spectral and intensity changes across the 2 h twilight dawn ramp). **D** α-opic lux ratio (relative to the maximum value) across 2 h dawn ramp, produced by LED powers in panel C. The ramp was achieved in a series of steps, indicated by dotted lines. **E** Light intensity (melanopic lux) across 2 h twilight ramp. **F** Daily light exposure (hrs) under simulated daylight and twilight ramped conditions. Light phase defined as start of ramp on to lights fully off. **G-I** Locomotor activity profiles with nestbox present, under simulated daylight and twilight ramped conditions, (**G**) across 24 h period (30 min bins), main cage, (**H**) across dusk (dusk indicated by dotted lines), 10 min bins, average of all three PIR sensors (**I**) across dawn (dawn indicated by dotted lines), 10 min bins, average of all three PIR sensors. **J** Locomotor activity onset (ZT) under simulated daylight and twilight ramped conditions. **K-M** Light environment sampling profiles under simulated daylight and twilight ramped conditions, (**K**) across 24 h period (30 min bins), (**L**) across dusk (dusk indicated by dotted lines), 10 min bins. **M)** across dawn (dawn indicated by dotted lines), 10 min bins. **N** Light environment sampling onset (ZT) under simulated daylight and twilight ramped conditions. **H,L** Data is plotted for ZT11-15, but statistics performed on ZT12-14 only. **L,M** Data is plotted for ZT21-1, but statistics performed on ZT22-24 only. **G-I,K-M** White and black bar shows timing of light and dark, respectively. ZT refers to “zeitgeber time” – with ZT0 defined as lights fully on. All results reported as mean across mice and days, ± SEM. Red squares indicate significant post hoc differences (**G**-**I**,**K**-**M**). ***p < 0.001, **p < 0.01, *p < 0.05 between condition comparisons (F,J,N)

We simulated spectral changes occurring at civil twilight (Fig. 5A) across the 2 h dawn and dusk ramp, in addition to changes in intensity (we referred to this condition as ‘twilight ramp’; Fig. 5B-E). The twilight ramp condition is matched for changes in melanopic lux to the simulated daylight ramp condition, across which spectrum remains constant (Figs. 4B, 5E). Therefore, the twilight and simulated daylight ramp conditions differ only in spectral composition at dawn and dusk. Our previous data also demonstrate that the most significant behavioural changes occur across these periods [12]. As such, dawn (ZT22-24) and dusk (ZT12-14) were analysed at a higher temporal resolution and averaged across all three PIR sensors to account for locomotor activity within the nestbox.

Mice exposed themselves to significantly more light under the twilight condition compared to the simulated daylight condition, by an average of 0.5h (Fig. 5F) [p = 0.0029, Wilcoxon signed rank test]. This increase in daily light exposure is likely to result from higher levels of main cage locomotor activity at dusk under the twilight condition (Fig. 5G,H). Whilst no main effect of condition on the overall daily profile of main cage locomotor activity was observed (Fig. 5G), [main effect of time, F(1.7,19.1) = 35.0, *p* < 0.0001; main effect of condition, F(1,11) = 2.2, *p* = 0.1630; time x condition interaction, F(3.7, 40.6) = 2.1, *p* = 0.1078; two-way repeated measures repeated measures ANOVA], locomotor activity was significantly higher across dusk (ZT12-14) under the twilight ramp compared to the simulated daylight ramp condition (Fig. 5H) [main effect of time, F(2.7,29.6) = 18.5, *p* < 0.0001; main effect of condition, F(1,11) = 11.4, *p* = 0.0062; time x condition, F(1.9,21.3) = 4.1, *p* = 0.0314; two-way repeated measures repeated measures ANOVA]. There were significant post-hoc differences at ZT13.2 and ZT13.3 (post-hoc Bonferroni test) – with activity levels being 1.7 × higher under the twilight ramp than the simulated daylight ramp at ZT13.3. However, there was no significant effect of condition on locomotor activity at dawn (Fig. 5I), [main effect of time, F(2.9,31.2) = 52.0, *p* < 0.0001; no main effect of condition, F(1,11) = 0.9, *p* = 0.3669; no time x condition interaction, F(3.3,36.2) = 0.7, *p* = 0.5467; two-way repeated measures repeated measures ANOVA]. Accordingly, activity onset was significantly earlier under twilight than the simulated daylight condition by 0.5h (Fig. 5J) [*p* = 0.0156, Wilcoxon signed rank test]. Mouse 3 free-ran under the twilight ramp condition and was removed from this analysis.

Light environment sampling behaviour showed similar differences between twilight and simulated daylight ramp conditions as locomotor activity. There was no main effect of condition on the overall daily profile of light environment sampling behaviour (Fig. 5K) [main effect of time, F(3.8,42.0) = 10.4, *p* < 0.0001; no main effect of condition, F(1,11) = 2.4, *p* = 0.1509; no time x condition interaction, F(5.4,59.2) = 1.8, *p* = 0.1263; two-way repeated measures ANOVA]. However, there was a significant difference in the pattern of light environment sampling behaviour across dusk between conditions (Fig. 5L) [main effect of time, F(3.6,39.5) = 13.7, *p* < 0.0001; main effect of condition, F(1,11) = 4.0, *p* = 0.0723; time x condition interaction, F(5.1,56.5) = 4.6, *p* = 0.0012; two-way repeated measures ANOVA]. Significant post-hoc differences were observed at ZT13.2 and ZT13.8 (post-hoc Bonferroni test) – with light environment sampling levels being 2.2 × higher under the twilight ramp than simulated daylight ramp at ZT13.8 (Fig. 5L). Accordingly, the peak in light environment sampling at dusk, or light environment sampling ‘onset’, occurs significantly earlier under the twilight ramp than under the simulated daylight ramp, by 1.1h (Fig. 5N) [t(10) = 4.6, *p* = 0.0010; paired t-test]. However, at dawn there was no significant difference in the pattern of light environment sampling behaviour across time between conditions (Fig. 5M), [main effect of time, F(3.8,41.9) = 8.5, *p* < 0.0001; main effect of condition, F(1,11) = 0.8, *p* = 0.3883; time x condition, F(2.9,32.1) = 1.9, *p* = 0.1470; two-way repeated measures ANOVA]. In summary, locomotor activity and light environment sampling behaviour are significantly higher and start earlier at dusk, but not dawn, under twilight spectral changes. This suggests that changes in spectral composition may be important for activity onset, but not offset.

## Discussion

To further our understanding of the regulation of light exposure and subsequent photoentrainment in the natural environment, we exposed mice to simulated daylight and twilight in the presence of a dark nestbox. Whole cage locomotor activity monitoring enabled quantification of light sampling behaviour, decision making (via assessment of ‘go’ or ‘no go’ movements from the atrium) and circadian entrainment [12]. We demonstrate that gradual changes in intensity are essential for driving crepuscular light sampling behaviour [12], whilst spectral changes at twilight [7] modulate the timing of behaviour at dusk.

As shown previously [12], light sampling is consistently higher at dawn than dusk (Figs. 4C, 5K). Dawn may be a more salient cue than dusk, since rising light levels are increasingly aversive to mice, generating a preference for shelter [60]. This could result in higher levels of light sampling behaviour at dawn as animals forage and explore before light levels become too aversive [12]. However, despite light sampling being higher at dawn than dusk, introducing natural changes in spectrum at twilight significantly affected behaviour at dusk (Fig. 5H,L) but not dawn (Fig. 5I,M). Specifically, the onset of locomotor activity at dusk was advanced by 0.5h under the twilight ramp (Fig. 5H) and light sampling behaviour was advanced by 1.1h (Fig. 5L). Since activity offset is known to be more variable in mice than activity onset [22, 59] we may be unable to detect behavioural differences under spectral changes at dawn due to higher levels of baseline variation. Alternatively, photoreceptor involvement in the detection of dawn and dusk may differ. It is clear how these tasks would differ under standard laboratory conditions—dusk requires the detection of decreasing light levels by a light-adapted retina (more suited to cones), whilst dawn requires detecting low, increasing light levels by a dark-adapted retina (more suited to rods). However, the presence of a nestbox will alter the photosensory task of detecting dawn and dusk. It may even make them more similar, since daytime nestbox use will promote retinal dark-adaptation at dusk. Our data shows the extent of daytime nestbox use to vary between individuals (Fig.S1A), which will generate different levels of retinal dark-adaptation. Mice spending more time in the main cage during the light phase may sample less, or later, since their retinas will be less sensitive (more light adapted). This could explain the lower average peak in light sampling events at dusk than dawn (Figs. 4C, 5K). Specifically, a nestbox may promote a role for rods at twilight; since although rod responses will still saturate, bleaching is less likely to occur compared to under standard laboratory conditions. Equally, the greater temporal contrast of intermittent light exposure in the presence of a nestbox may increase the role of cones [20, 61]; although this is dependent on the length of light sampling events. Daily variations in photoreceptor function resulting from the retinal clock will add further complexity to photoreceptor contribution [62, 63]. The study of photoreceptor knockout models under these naturalistic twilights could provide further insight [19]; although the use of such models may be confounded by the impact of developmental reorganisation.

Comparison of twilight conditions against standard 12:12 LD cycles are complicated. Adding a 2 h ramped change of lighting at dawn and dusk to the existing 12 h of lighting will effectively extend the photoperiod. This will lead to changes in activity onset and offset in comparison to a square-wave 12:12 LD cycle (Fig. 2B). However, this alone cannot explain the changes in light sampling behaviour, with prominent crepuscular peaks ( [12] and replicated here in Fig. 2C). These appear to be a response to gradually changing light intensities, potentially creating conflicting signals to retreat or forage. Furthermore, changes in photoperiod cannot explain any differences due to daylight or twilight simulation we show here (Figs. 3, 4, and 5), as all these studies are compared against conditions of identical photoperiod.

Collectively, our data suggest that the colour of light at twilight has a significant effect upon mouse behaviour. Spectral changes at twilight must therefore be detected by the retina before being communicated to the brain to influence behaviour—either via the circadian system [15, 19] or through the direct alteration of activity by light, known as masking [46, 64]. Due to the dorsal–ventral gradient in cone opsins, cone-cone opponency in mice is very rare (~ 3% of RGCs) ( [65, 66]. Instead, murine colour vision is thought to be driven by rod-cone opponency in the ventral retina. A recently identified subset of RGCs, referred to as JAMB cells, were shown to have an inhibitory S-cone centre and an excitatory rod surround [66, 67]. As such, they are strong contenders to act as twilight detectors, since they function under low light intensities and respond to UV light, which is enriched at twilight [7].

The differences in timing of behaviour observed in our data under naturalistic light environments could be driven by the circadian system, since a subset of mouse SCN neurons are known to be sensitive to colour [19]. However, it remains an open question as to how chromatic input reaches the SCN. The M1 pRGC subclass are the primary conduit of both intrinsic and extrinsic signals to the SCN via the retinohypothalamic tract (RHT) [68, 69], but do not exhibit cone-mediated colour opponent responses [66, 70]. Conversely, although M5 rodent cells receive cone opponent signals, these are not thought to project directly to the SCN [71]. Approximately 25% of SCN neurons exhibit opponent responses to selective stimulation of S-cones and M-cones, with the majority demonstrating blueON:yellowOFF responses [19]. This is much higher than the 3% of RGCs found to display cone-cone opponency ( [65, 66], suggesting that cone opponent responses in the SCN could be an emergent feature, rather than derived at the level of the retina [66]. The effects of spectral cues at twilight on body temperature rhythms were abolished in mice lacking cones [19], suggesting a role for cones as an origin of colour information in the SCN [15, 16] – perhaps via the rod-cone opponency of JAMB cells [66, 67]. Alternatively, it is possible that separate inhibitory (via recently identified GABAergic pRGCs projecting from M-cone dominated areas of the retina [72] and excitatory pRGCs (via glutamatergic pRGCs projecting from S-cone dominated areas of the retina) could provide convergent input to the SCN [65, 66, 73]. Therefore, whilst studies using 12:12h LD cycles of white light suggest greater role for rods and melanopsin in photoentrainment [14, 74, 75], under natural conditions where spectrum is variable [7] and temporal contrast is higher due to light sampling behaviour [12, 20], cones are likely to play a more important role in photoentrainment.

Circadian sensitivity to colour could provide a selective advantage by enabling more accurate tracking of twilight, and subsequent entrainment [23]. Mouland et al., 2019 [15] demonstrated that the mouse circadian system is less responsive to blue light than yellow light of equivalent brightness. This differential sensitivity could allow for the stages of twilight to be distinguished more accurately. For example, at the start of dawn light appears blue enriched and is also dimmer, resulting in weaker circadian responses. Whilst at the end of dawn, light appears more yellow and is also brighter, resulting in stronger circadian responses. The constriction of the mouse pupil in response to S-cone activation could exacerbate this difference [43]; although the low light intensities of natural twilight compared to those required for pupillary light constriction may minimise this effect. The elimination of shared noise by spectral opponency could also promote more accurate tracking of twilight when stochastic variations in light levels (e.g. from cloud cover, or movement in and out of shade) make absolute irradiance a less reliable zeitgeber [15, 18, 27, 76]. Similarly, retinal light adaptation and even rod-bleaching may make it harder to respond to decreasing light intensity at dusk, rendering changes in colour a useful additional time cue. Tracking twilight using colour may therefore not only help improve the accuracy of time of day detection, but also of time of year – since this is based on the detection of photoperiod [27].

Although circadian sensitivity to colour has been previously demonstrated in mammals [15, 19], due to maintaining all animals under entrained conditions in our study, conclusions about core circadian rhythms are challenging. Therefore, the effects of colour on behaviour observed in our data could originate from direct effects of light on activity. It is well established that bright light suppresses activity in nocturnal rodents (negative masking) whilst dim light promotes activity (positive masking) [64, 77, 78]. Melanopsin has been suggested as the major regulator of negative masking, whilst positive masking appears to require classical photoreceptors [77–81]. The role of colour in acute responses to light has only recently been investigated [46, 82]. Tamayo et al., 2023 [46] demonstrated that selective activation of S-cone irradiance (similar to that experienced at twilight) results in increased activity and light-seeking behaviour in mice. This phenomenon was found to be spectrally-opponent, with longer-wavelength sensitive cones resulting in the opposite effect [46]. This aligns with the increased light sampling behaviour (Fig. 5L,N) and earlier activity onset (Fig. 5J) observed at dusk under blue-enriched twilight ramps in our data. Furthermore, the clear increase in locomotor activity under the twilight ramp occurs ~ 40 min into the dusk ramp (Fig. 5H), coinciding with the point at which the ratio of S-cone opic lux increases most steeply (Fig. 5D). At this time point, there are 12.8 log quanta (88 alpha-opic lux) available to S-cones and 13.6 log quanta (89 alpha-opic lux) available to M-cones (Additional file 2) – both within the ~ 10 to 15 log quanta sensitivity range of cones [45, 83]. Further experiments to separate the acute and circadian effects of light would be informative. For example, studying the phase angle of entrainment in constant darkness following twilight ramps may enable the effects of masking to be addressed.

It is unclear how far all these findings may extend to humans, especially as temporal niche may alter the response to spectral cues [84, 85]. The literature is also conflicted as to how colour influences other SCN-driven responses to light in humans such as melatonin suppression [86, 87]. However, the presence of colour opponency in primate pRGCs [83] indicate that colour could have a modulatory effect on human circadian sensitivity ( [18]). Studying circadian responses to more complex visual cues likely to be encountered in the real world may reveal differences in photoreceptor contributions [88]. If this is indeed the case, it would raise important issues with regard to lighting design [27, 89–91]. In addition, our data raises the possibility that changes in daytime light exposure could be altering sleep homeostatic pressure, leading to changes in subsequent sleep behaviour, as has been observed in humans [92, 93].

## Conclusions

Collectively, our data demonstrate that a gradual change in light intensity is critical for driving increased crepuscular light sampling behaviour in mice [12] and that naturally-occurring spectral changes across twilight alter the timing of both locomotor activity and light sampling. Recent guidance on laboratory mouse lighting suggest housing animals under more natural lighting conditions and giving animals the opportunity to avoid light [31]. Our data provide the first evidence that such housing conditions alter behaviour, particularly when dynamic changes at dawn and dusk are used. Studying laboratory mice under more naturalistic housing conditions may benefit both animal welfare and scientific reproducibility.

## Methods

### Animals and housing conditions

A cohort of 12 C57BL/6J mice (6 female and 6 male; Inotiv, Blackthorn, UK, RRID: IMSR_ JAX:000664) aged ~ 8 weeks at the start of the control week were used. This strain was chosen to address how wildtype mice respond to light, in order to be of greatest relevance for animals used by the majority of researchers. All animals were singly housed with ad libitum access to food and water (located in the main cage), and a small amount of sizzlenest (Sizzlenest, Datesand; UK) was present throughout the experiment. Cages were placed in light-tight chambers (LTC) equipped with multiple light-emitting diodes (LEDs) (details outlined in the experimental design and visual stimuli section of the methods). The animal holding room was maintained at 19–21°C and at 45–65% ± 10% humidity, as per UK regulatory standards.

### Experimental design

Stimuli complexity was introduced gradually across the experiment, via a series of different conditions. All animals received the same set of conditions, in the same order (Table 1). All LD cycles were reversed, to minimise disruption from daily welfare checks. Firstly, animals were habituated to the home cage under a 12:12h LD cycle of 200 photopic lux (170 melanopic lux) of a cool-white LED (Fig. 1A, blue line). This was generated by WiFi controlled cool-white (4500K CCT) LEDs (LIFX light strip; LIFX, Cremorne, Australia), the SPD of which consisted of a high, narrow peak at ~ 450nm and a lower, broader peak at ~ 560nm (Fig. 1A, blue line), measured using a calibrated Ocean Optics USB2000 + Spectrophotometer (Ocean Insight, Orlando, FL, United States). Light conditions remained constant whilst 1 week of control recordings took place (condition 1). A nestbox was then added to the home cage (condition 2), which was present for the remainder of the experiment. The 12:12h LD cycle of white LED was then replaced by a 12:12h LD cycle of simulated daylight (condition 3, ‘daylight’), before being replaced by a 12:2:8:2h ramped LD cycle of white LED (condition 4, ‘white LED ramp’) and simulated daylight (condition 5, ‘daylight ramp’). Finally, spectral changes associated with civil twilight were introduced across the LD cycle ramp (condition 6, ‘twilight ramp’). The ramps in conditions 5 and 6 were matched for changes in intensity (melanopic lux). However, whilst the spectral ratio across the ramp remained constant in condition 5, they simulated civil twilight in condition 6. Conditions 2 and 4 had three days of habituation at the beginning, to allow for habituation to the nestbox and a ramped light dark cycle, as previous data showed that activity patterns stabilised after this period [12]. Only the last week of recordings in these conditions were analysed.

**Table 1:** Series of experimental conditions. Italics indicates aspect of experimental design that is changing with each condition. ‘mel lux’ refers to melanopic lux. ‘LD cycle’ refers to light:dark cycle. ‘hr’ refers to hour

Condition	Day	Purpose	Nestbox	Light environment	LD cycle
N/A	1–12	Habituation	No	169.7 mel lux white LED	Reverse 12:12h
1	12–19	Control	No	169.7 mel lux white LED	Reverse 12:12h
2	19–29	Experimental	*Yes*	169.7 mel lux white LED	Reverse 12:12h
3 (‘daylight’)	29–36	Experimental	Yes	*169.7 mel lux simulated daylight*	Reverse 12:12h
4 (‘white LED ramp’)	36–46	Experimental	Yes	169.7 mel lux white LED, followed by intensity only ramp	*Reverse 12:2:8:2h*
5 (‘daylight ramp’)	46–53	Experimental	Yes	*169.7 mel lux simulated daylight, followed by intensity only ramp*	Reverse 12:2:8:2h
6 (‘twilight ramp’)	53–60	Experimental	Yes	*169.7 mel lux simulated daylight, followed by a ramp with twilight intensity and spectral changes*	Reverse 12:2:8:2h

### Locomotor activity monitoring

To record locomotor activity a passive infrared sensor (PIR) was fitted 22 cm above each cage [94]. The PIR sensors record movement as a binary measurement every 10ms and combines this data across 1 s bins, outputting a percentage activation of the sensor across every 1 s epoch. From condition 2 onwards, a dark nestbox was placed into each cage – following the same protocol as [12]. In brief, the nestboxes (Fig. 1C) had two internal sections (Fig. 1D) – a dark nesting section at the back, and a lighter atrium section at the front. The nesting section had a light level of < 0.1 photopic lux, whilst the atrium section ranged from < 0.1 to 0.4 photopic lux, as measured by an XL-500 BLE Spectroradiometer (dynamic range = 0.1 to 40,000 photopic lux; NanoLambda, Korea) (Fig. 1E). Both sections were fitted with PIRs collecting locomotor activity data every 1 s, as in the main cage, and mice could move freely between the main cage, the atrium and nesting section. Food and water were located in the main cage. Locomotor activity was defined as movement in the main cage, outside of the nestbox. Light sampling behaviour was defined as a movement within the nestbox, from the dark nest to atrium compartment. From the atrium, the animal can sample the external light environment, in a similar way to at a burrow entrance. Under this definition, light sampling behaviour can occur across the 24 h period, including during the dark phase when there is no light to sample. For this reason, it is perhaps more accurate to describe it as ‘light environment sampling’ behaviour where the light environment could be light or dark [12]. For simplicity, these terms will be used interchangeably. The LTC light schedule was confirmed using a light-dependent resistor (LDR). For more details see [12].

### Data processing

Raw PIR data was processed in MATLAB (v.R2024a), ImageJ (v.1.53a, using the Actogram J plugin [95]) and Excel (v.2310). Data was processed using methods described in [12]. Given the amount of data and processing involved, these are replicated here for clarity and reproducibility.

### Locomotor activity profiles

To generate daily locomotor activity profiles for each experimental condition, raw main cage PIR activity and LDR data was averaged into 30 min bins, starting at ZT0 for 7 consecutive days (ZT = zeitgeber time; ZT0 = lights fully on, ZT12 = 12 h after ZT0). The activity level for each time bin represents the average level of activity from that time point to 30 min after that time point i.e. ZT0 represents the average activity from ZT0 to ZT0.5.

### Location and light exposure

A MATLAB function *(location_finder.m)* was written to calculate the location (cage, atrium or nest) of each mouse at each 1 s time point. This function filled in the location of the mouse using the 3 PIR channels of activity data (cage, atrium, nest) to account for the mouse being present but immobile in a location. If all PIR channels were reading 0, then it moved back rows until it hit a value of > 0 in one of the columns. A value of 1 was assigned to this channel. This produced a dataset for each mouse, where 1 equalled present and 0 equalled not present, across every second at all three locations (cage, atrium, nest). Using the location data, daily light exposure could subsequently be calculated. This was defined as the time spent (hrs) in the main cage during the light phase. For the control week this is automatically 12 h, as there was no nestbox available.

### Light environment sampling behaviour and decision making

Analysis of light environment sampling behaviour and decision making was also based on the location data. A MATLAB function *(simplify_columns.m)* was written which took the location data and generated a new matrix, to ensure that only one sensor was active at a time (if a mouse moved across the three PIR sensors within 1–3 s, then two or three sensors would show activity at each time point, due to sensor lag). If all three location columns equalled 0, three 0 s were assigned to the new matrix. If all columns were 1, a 1 was assigned to the atrium column and a 0 to the nest and cage (since the mouse is moving from the cage to the nest, through the atrium, or vice versa). If two columns equalled 1, then it moved up rows until one of the rows equalled 0. A 0 was assigned to this column and a 1 to the other column. A MATLAB function *(transitions.m)* was written in MATLAB to then take the simplified data and create a new matrix identifying light environment sampling behaviour (defined as a nest to atrium transition, and assigned as ‘1’ in the new matrix), followed by either entry to the cage (a “go” decision, assigned as ‘2’) or a return to the nest (a “no-go” decision, assigned as ‘3’).The sum of the “go” and “no go” transitions equalled the total number of light environment sampling events.

### Circadian parameters

Key circadian entrainment metrics were calculated as in Brown et al. (2019), using activity data from the main cage PIR sensor. MATLAB was used to calculate light phase activity, dark phase activity, relative amplitude, inter-daily stability and intra-daily variability. The chi-squared periodogram power (Qp) [96] and activity onsets were calculated using inbuilt functions in Actogram J [95].

### Visual stimuli

#### Light measurements

The traditional unit of light intensity, photopic lux, is based on human visual sensitivity. Therefore, lights differing in spectrum, but matched in brightness for a human observer, will appear differently to mice [31, 97]. Alpha-opic lux, weighted to each photoreceptor individually, is therefore necessary to study the effects of colour and irradiance independently in mice [97]. By allowing us to measure the activation of each photoreceptor type separately, alpha-opic lux is a useful tool for dissecting the role of different photoreceptors in regulating behaviour and physiology in wildtype mice [15, 19, 98–100].

### Simulating daylight

Multichannel LEDs (Fig. 1B), comprised of a violet LED strip (λmax = 405nm; custom built by the Oxford University Physics Workshop, and controlled via a custom LabView script), blue LED strip (λmax = 450nm; WiFi controlled, LIFX) and green LED strip (λmax = 520nm; WiFi controlled, LIFX), were used to simulate the experience of daylight on the mouse retina. Daylight was defined as the light environment measured at 24.1 degrees above the horizon on 19/07/2014 at 18:08:44 (Additional file 3: Table S2; [7] – measured in Cherry Springs State Park, Pennsylvania, US; a certified International Dark Sky Park). This measurement was selected as it started at a shorter wavelength (340nm) than the other daylight measurements, which started at 360nm. Therefore it matched the equivalent daylight illuminance (EDI) definition of daylight (equal alpha-opic lux) more closely [31]. However, since the measurements only started at 340nm, S-cone opic lux was still lower (156 lx) than alpha-opic lux values for the other photoreceptors (~ 169 lx) (Fig. 3B). This daylight SPD was converted to 5nm bins from 300-780nm by linear interpolation, and normalised to the summated total to produce a relative SPD (RSPD) (Fig. 3A, ‘daylight’). The Rodent Toolbox (Additional file 4) was used to calculate the α-opic lux (Fig. 3B, ‘daylight’) and log quanta (Additional file 3: Table S1, ‘daylight’) available to each mouse photopigment, produced by 170 melanopic lux of the daylight RSPD – to match the 170 melanopic lux produced by 200 photopic lux of the cool-white LED used in the habituation and control conditions.

The RSPDs of the three LED strips (Fig. 1B) were measured using a calibrated Ocean Optics USB2000 + Spectrophotometer (Ocean Insight, Oxford, United Kingdom) and the sum used as the input for the Rodent Toolbox which had been modified to calculate metamers (Additional file 5). The solver optimisation function in Excel (v2309) was used to calculate the power (µW/cm^2^/s) required by each LED strip to produce the equivalent α-opic lux values as the daylight measurement. All LEDs were set to initial values of 10 µW/cm^2^/s before the solver function was used. The following LED powers were calculated: violet (43.2 µW/cm^2^/s), blue (3.3 µW/cm^2^/s) and green (22.3 µW/cm^2^/s) (Fig. 3A); producing α-opic lux values all within 0.4% of the target values (1dp) (Fig. 3B), and log quanta values that matched target values to 1dp (Table S1). The multichannel LEDs were set to the corresponding power using a power metre (PM160 wireless power metre, Thor Labs). α-opic lux values were confirmed to be within 2% of the target values, and log quanta values matched target values to 1dp, as measured using a calibrated Ocean Optics USB2000 + Spectrophotometer and calculated using the Rodent Toolbox (Additional file 4).

Condition 3 and 5 both used this simulated daylight environment (Table 1). Condition 3 used a reverse 12:12h LD cycle of 170 melanopic lux of simulated daylight. Condition 5 used a reverse 12:2:8:2h LD cycle of 170 melanopic lux of simulated daylight during the light phase (ZT0-ZT12), and a series of step changes in light intensity across the dawn/dusk ramps (Fig. 4A,B), but with spectral ratios remaining equal to that produced by simulated daylight (Fig. 3B). The timings of the 12:2:8:2h ramped LD cycle were based on the length of daylight and twilight naturally occurring at the spring and vernal equinoxes in Oxford, UK (sourced from [101]). The equinox LD cycle was used since it is an intermediate LD cycle, with the length of the light phase being in-between those of the summer and winter solstices. Validation measurements were taken at 0, 56 and 92 min into the 2 h dawn/dusk ramp – alpha opic lux values were within 9% of target values and log quanta values were within 1dp of target values (Additional file 6). Steps were necessary due to the technical limitations of the violet LED strip which ramped in intensity via a set series of steps (Additional file 7). The overall dawn/dusk ramps were exponential, with light intensity at 1 h into the ramp measuring 46.8 photopic lux (68.4 S-opic, 74.9 melanopic, 74.8 rhodopic, 74.2 M-opic lux) (Fig. 4B). The white LED ramp in condition 4 (Table 1) followed the same overall step changes in light intensity as the simulated daylight ramp, with light intensity defined by melanopic lux (data not presented). The multichannel LEDs were set to the corresponding power for each step of the ramp using a power metre (PM160 wireless power metre, Thor Labs).

### Simulating twilight

Condition 6 (Table 1) used a reverse 12:2:8:2h light dark cycle of 170 melanopic lux of simulated daylight during the light phase (Fig. 3A), and an exponential change in light intensity across the 2 h ramp via a series of steps (Fig. 5E) which matched the intensity changes in condition 5 (Fig. 4B), as quantified by melanopic lux. This change in intensity occurred in combination with spectral changes associated with civil twilight (0 to −6 degrees solar elevation). Civil twilight was selected as this is the stage of twilight where the largest spectral changes occur (Fig. 5A), with spectral changes stabilising in nautical (−6 to −12 degrees solar elevation) and the signal to noise ratio becoming too low across astronomical twilight (−12 to −18 degrees solar elevation) (Additional file 8: Fig.S2). The SPDs for each 1 degree increment of solar elevation from 0 to −6 degrees, as measured on 30/06/2014 between 20:28:05 and 21:06:06 (S2; [7], were converted into 5nm bins from 300-780nm, and normalised to the summated total to produce RSPDs (Additional file 9). The Rodent Toolbox (Additional file 4) was used to calculate the ratio of α-opic lux (relative to the maximum value) available to each mouse photopigment produced by the RSPD of each degree of solar elevation across civil twilight. This method enabled us to simplify the spectral changes observed across twilight (Fig. 5A) to relevant changes in α-opic lux experienced by the mouse retina across civil twilight (Fig. 5B).

0 and −1 degrees of solar elevation produced the same α-opic lux ratio (Fig. 5B). Therefore, 0 degrees was removed from the simulation. Each α-opic lux ratio for the remaining degrees of solar elevation (−1 to −6 in 1 degree increments) were assigned to each of the ramp intensity steps (simulating the −1 degrees α-opic lux ratio at the brightest step, down to −6 degrees α-opic lux ratio for the dimmest step). The RSPDs for −1 to −6 degrees were input into the Rodent Toolbox and corrected to produce the appropriate melanopic lux to match each step change in intensity across the ramp in condition 5. The α-opic lux values produced were then used as target values in the solver optimisation function in Excel (v2309) to calculate the power (µW/cm^2^/s) required by each LED (Fig. 5C). All LEDs were set to a starting power of 10 µW/cm^2^/s before the solver function was used. The α-opic values produced by each stage of the twilight ramp (Fig. 5D) were within 0.9% of the target values (Fig. 5B), and log quanta values were within 1dp of target values (Additional file 3: Table S2). The overall ramp was exponential, with light intensity at 1 h into the ramp measuring 38.8 photopic lux (74.3 S-opic, 74.9 melanopic, 70.7 rhodopic, 68.2 M-opic lux) (Fig. 5E). The multichannel LEDs were set to the corresponding power for each step (Fig. 5C) using a power metre (wireless power metre, Thor Labs). Validation measurements were taken at minute 0, 56 and 92 into the 2 h ramp. α-opic lux values were confirmed to be within 10% of the target values, and log quanta values matched target values to 1dp, as measured using a calibrated Ocean Optics USB2000 + Spectrophotometer and calculated using the Rodent Toolbox (Additional file 4).

### Statistical analysis

Statistical analysis and data visualisation were performed in MATLAB and Prism Graph-pad (v.9.5.0 (730)). α = 0.05 was adopted in all analyses. All locomotor activity, light environment sampling and decision making daily profiles are visualised in 30 min bins, unless otherwise stated (Fig. 5H,I,L,M). Any animals that did not routinely use the nestbox were removed from the analysis (mouse 1 was excluded from light exposure analyses in Fig. 2 and Fig. 3; and mouse 8 was excluded from light exposure analyses in Fig. 4 and Fig. 5). Mouse 3 free-ran under the twilight condition and was subsequently removed from the twilight condition analysis (Fig. 5). The Greenhouse–Geisser correction was performed with all ANOVAs, and corrected degrees of freedom reported, unless otherwise stated. A post-hoc Tukey test was used when all pairwise comparisons were desired, whilst a post-hoc Bonferroni test was used for a specific comparison between the control and experimental treatments. Where comparisons were made to confirm the presence of dawn/dusk changes in light sampling behaviour (Fig. 2C and Additional file 10: Fig.S3), Fisher’s LSD post hoc tests were used. Further details on statistical tests used for each dataset are reported in the results section. Locomotor activity and light environment sampling onset for every 24 h period for each mouse was calculated using Actogram J’s inbuilt function, which first smooths the data (using the standard deviation of a smoothing Gaussian distribution). Following this, activities are considered to be ‘active’ if they exceed the threshold of the median of all activity values, or all activity values excluding zero (best fit assessed visually to ensure no systematic bias) [95].

## Supplementary Information

Additional file 1. Supplementary figure 1 [Fig.S1: (A) Daily light exposure (hrs) across individuals under the no-nestbox control condition (dark blue) and nestbox conditions (light blue), under a square-wave 12:12hr white LED LD cycle. Daily light exposure under the control condition is automatically 12hrs, since there is no nestbox present. (B) Daily light exposure (hrs) under the nestbox condition (square-wave 12:12hr white LED LD cycle) averaged across individuals, by sex. All data reported as mean +/- SEM. **** *p*<0.0001].

Additional file 2. Validation daylight intensity-only ramp measurements.

Additional file 3. Supplementary table 1 and 2 [Table S1: log quanta (1dp) available to each photoreceptor under daylight and simulated daylight. Table S2: log quanta (1dp) available to each photoreceptor across civil twilight (corrected for changes in melanopic lux occurring across the daylight intensity-only ramp – condition 5) and simulated twilight condition].

Additional file 4. Rodent toolbox, used to calculate alpha-opic lux and log quanta from an SPD.

Additional file 5. Rodent toolbox amended so that the input is the sum of the multichannel LEDs RSPDs. Used to calculate the power of each LED required to achieve target alpha-opic lux.

Additional file 6. Daylight intensity-only ramp (condition 5) calculations.

Additional file 7. Validation daylight measurement - SPD of 169.7 melanopic lux of simulated daylight.

Additional file 8. Supplementary figure 2 [Fig.S2: Spectral changes (RSPDs) across nautical (A) and astronomical (B) twilight in increments of 2 degrees of solar elevation, relative to the horizon (data from Spitschan et al, 2016)].

Additional file 9. Selected SPDs taken from Spitschan et al, 2016. Single daylight measurement and civil twilight measurements.

Additional file 10. Supplementary figure 3 [Fig.S3: Daily light environment sampling profile under a square-wave LD cycle (blue) and a ramped LD cycle (green) of simulated daylight. Square-wave LD cycle refers to 12:12hr LD. Ramped LD cycle refers to 12:2:8:2hr LD cycle. White, grey and black bar shows timing of light, light ramp and dark, respectively. All results reported as mean across mice and days,+/- SEM. Two-way repeated measures ANOVA [main effect of time, F(5.3,58.7) = 11.1, *p*<0.001; main effect of condition, F(1,11) = 6.5, *p* = 0.0267; main interaction effect, F(5.8,63.5) = 4.6, *p* = 0.0007]. Red squares indicate post hoc differences between groups. Post hoc differences observed at dawn (ZT13.5;*p* = 0.0046) and dusk (ZT22, *p* = 0.0004); Fishers LSD test].

## Data Availability

The datasets used and/or analysed during the current study are available from the corresponding author on reasonable request.
