# Supplementary figures and images for: Simulated natural daylight and twilight modulate activity and light sampling behaviour in mice
Source: BMC Biol. 2026 Jan 22;24:40. doi: 10.1186/s12915-026-02517-7 (PMC12911055; doi:10.1186/s12915-026-02517-7)

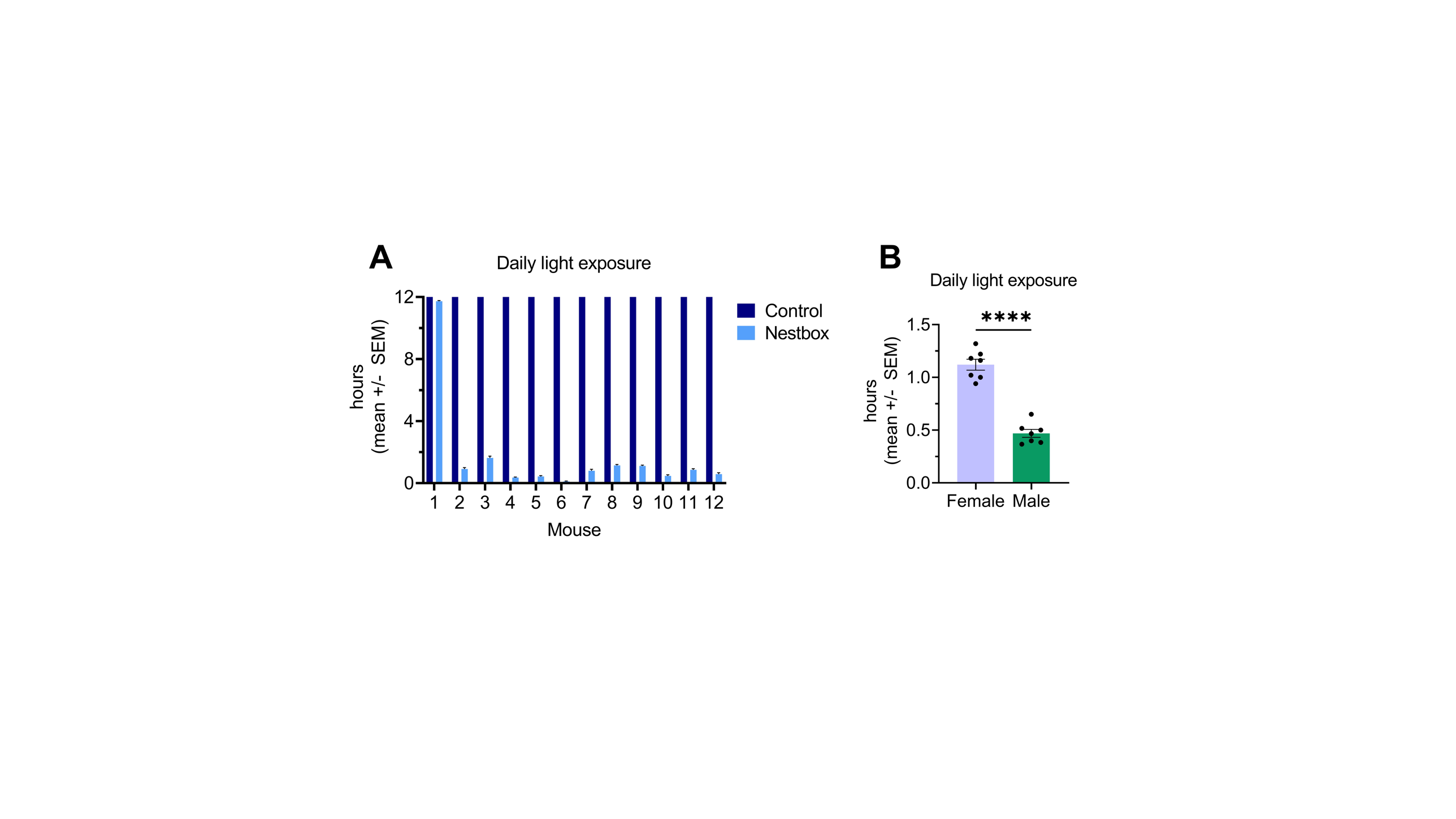

Supplement: Supplementary file 1 — Additional file 1. Supplementary figure 1 [Fig.S1: (A) Daily light exposure (hrs) across individuals under the no-nestbox control condition (dark blue) and nestbox conditions (light blue), under a square-wave 12:12hr white LED LD cycle. Daily light exposure under the control condition is automatically 12hrs, since there is no nestbox present. (B) Daily light exposure (hrs) under the nestbox condition (square-wave 12:12hr white LED LD cycle) averaged across individuals, by sex. All data reported as mean +/- SEM. **** p<0.0001]. [file 12915_2026_2517_MOESM1_ESM.jpg]

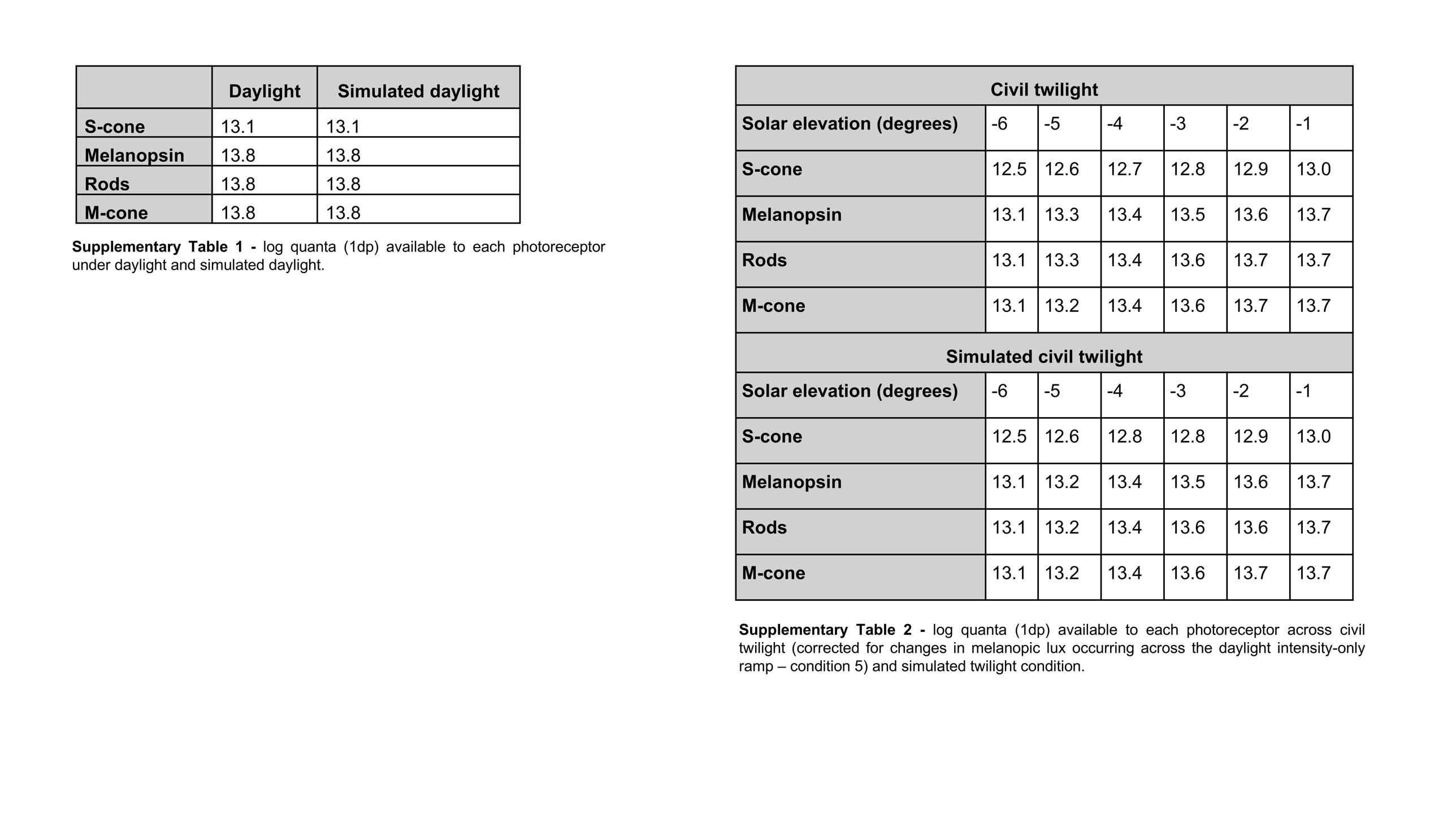

Supplement: Supplementary file 3 — Additional file 3. Supplementary table 1 and 2 [Table S1: log quanta (1dp) available to each photoreceptor under daylight and simulated daylight. Table S2: log quanta (1dp) available to each photoreceptor across civil twilight (corrected for changes in melanopic lux occurring across the daylight intensity-only ramp – condition 5) and simulated twilight condition]. [file 12915_2026_2517_MOESM3_ESM.jpg]

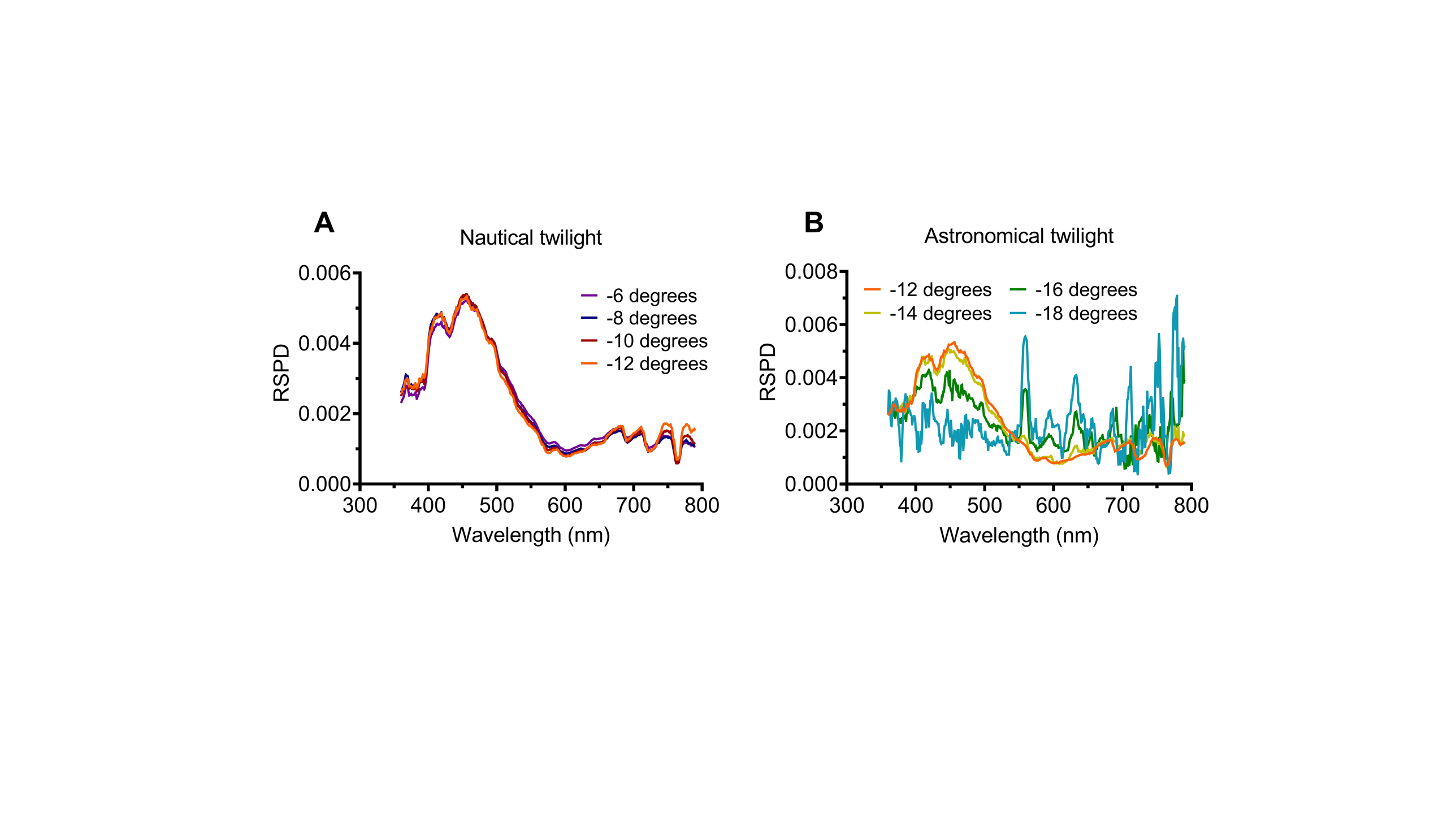

Supplement: Supplementary file 8 — Additional file 8. Supplementary figure 2 [Fig.S2: Spectral changes (RSPDs) across nautical (A) and astronomical (B) twilight in increments of 2 degrees of solar elevation, relative to the horizon (data from Spitschan et al, 2016)]. [file 12915_2026_2517_MOESM8_ESM.jpg]

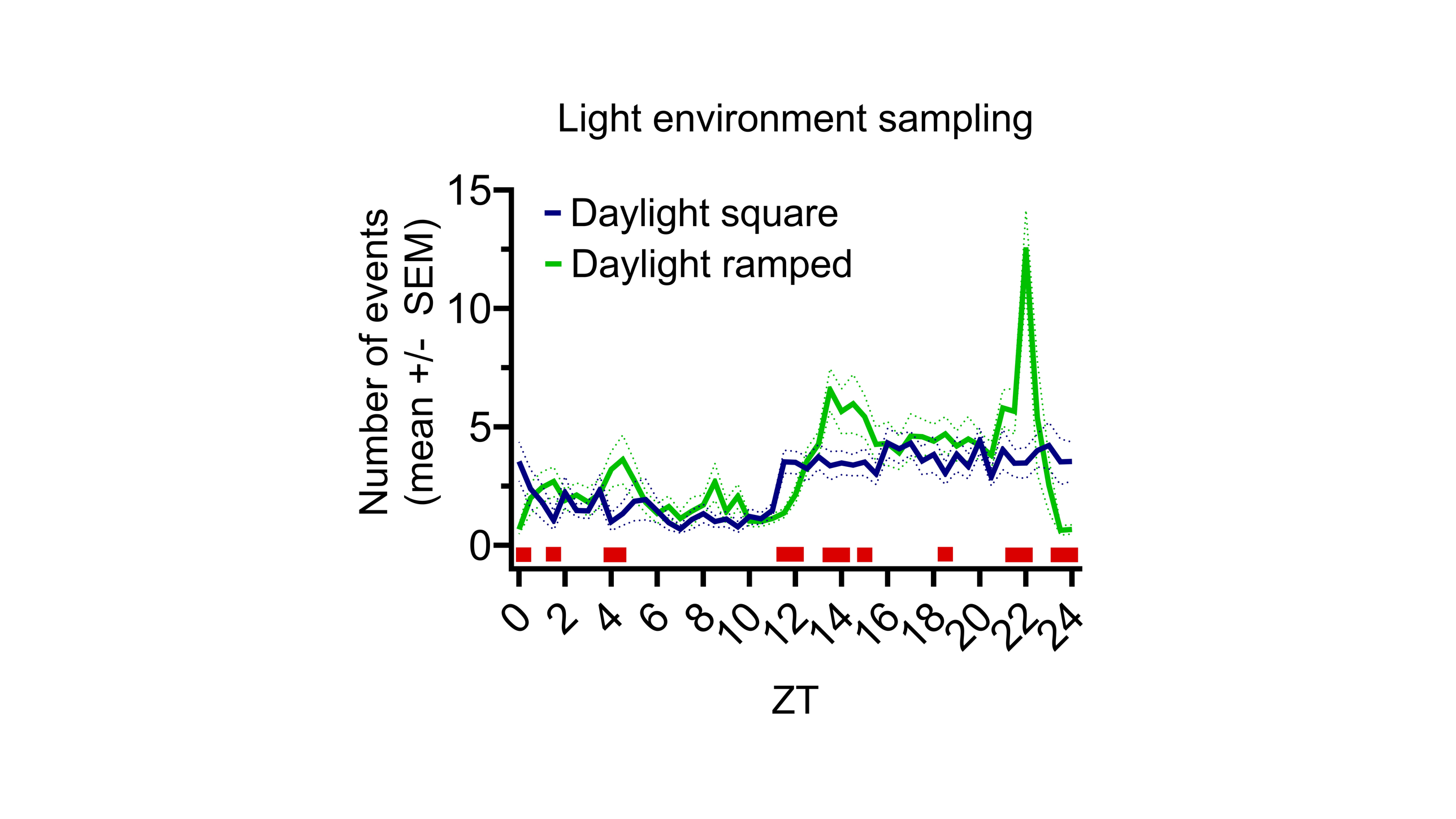

Supplement: Supplementary file 10 — Additional file 10. Supplementary figure 3 [Fig.S3: Daily light environment sampling profile under a square-wave LD cycle (blue) and a ramped LD cycle (green) of simulated daylight. Square-wave LD cycle refers to 12:12hr LD. Ramped LD cycle refers to 12:2:8:2hr LD cycle. White, grey and black bar shows timing of light, light ramp and dark, respectively. All results reported as mean across mice and days,+/- SEM. Two-way repeated measures ANOVA [main effect of time, F(5.3,58.7) = 11.1, p<0.001; main effect of condition, F(1,11) = 6.5, p = 0.0267; main interaction effect, F(5.8,63.5) = 4.6, p = 0.0007]. Red squares indicate post hoc differences between groups. Post hoc differences observed at dawn (ZT13.5;p = 0.0046) and dusk (ZT22, p = 0.0004); Fishers LSD test]. [file 12915_2026_2517_MOESM10_ESM.jpg]
